# Design of a Penta-Band Graphene-Based Terahertz Metamaterial Absorber with Fine Sensing Performance

**DOI:** 10.3390/mi14091802

**Published:** 2023-09-21

**Authors:** Runing Lai, Hao Chen, Zigang Zhou, Zao Yi, Bin Tang, Jing Chen, Yougen Yi, Chaojun Tang, Jianguo Zhang, Tangyou Sun

**Affiliations:** 1 Joint Laboratory for Extreme Conditions Matter Properties, Tianfu Institute of Research and Innovation, State Key Laboratory of Environmental Friendly Energy Materials, Key Laboratory of Manufacturing Process Testing Technology of Ministry of Education, Southwest University of Science and Technology, Mianyang 621010, China; lrn3167160774@163.com (R.L.); chenhaoswustedu@163.com (H.C.); zhouzigang1973@163.com (Z.Z.); 2 School of Chemistry and Chemical Engineering, Jishou University, Jishou 416000, China; 3 School of Microelectronics and Control Engineering, Changzhou University, Changzhou 213164, China; btang@cczu.edu.cn; 4 College of Electronic and Optical Engineering, Nanjing University of Posts and Telecommunications, Nanjing 210023, China; jchen@njupt.edu.cn; 5 College of Physics and Electronics, Central South University, Changsha 410083, China; yougenyi@csu.edu.cn; 6 College of Science, Zhejiang University of Technology, Hangzhou 310023, China; chaojuntang@126.com; 7 Department of Physics, Jinzhong University, Jinzhong 030619, China; phys.zhangjg@gmail.com; 8 Guangxi Key Laboratory of Precision Navigation Technology and Application, Guilin University of Electronic Technology, Guilin 541004, China

**Keywords:** terahertz, graphene, penta-band absorption, dynamic tunability, wide-angle absorption, high figure of merit

## Abstract

This paper presents a new theoretical proposal for a surface plasmon resonance (SPR) terahertz metamaterial absorber with five narrow absorption peaks. The overall structure comprises a sandwich stack consisting of a gold bottom layer, a silica medium, and a single-layer patterned graphene array on top. COMSOL simulation represents that the five absorption peaks under TE polarization are at *f*_I_ = 1.99 THz (95.82%), *f*_Ⅱ_ = 6.00 THz (98.47%), *f*_Ⅲ_ = 7.37 THz (98.72%), *f*_Ⅳ_ = 8.47 THz (99.87%), and *f*_V_ = 9.38 THz (97.20%), respectively, which is almost consistent with the absorption performance under TM polarization. In contrast to noble metal absorbers, its absorption rates and resonance frequencies can be dynamically regulated by controlling the Fermi level and relaxation time of graphene. In addition, the device can maintain high absorptivity at 0~50° in TE polarization and 0~40° in TM polarization. The maximum refractive index sensitivity can reach *S*_V_ = 1.75 THz/RIU, and the maximum figure of merit (FOM) can reach FOM_V_ = 12.774 RIU^−1^. In conclusion, our design has the properties of dynamic tunability, polarization independence, wide-incident-angle absorption, and fine refractive index sensitivity. We believe that the device has potential applications in photodetectors, active optoelectronic devices, sensors, and other related fields.

## 1. Introduction

Terahertz (THz) waves constitute a distinctive subset within the electromagnetic spectrum, spanning a frequency range of 0.1~10 THz (1 THz = 10^12^ Hz). This exceptional frequency band holds paramount significance across diverse domains, encompassing optical sensing [1], communication technology [2], biodetection [3], and optical stealth [4]. Its appeal emanates from its notable attributes, including low energy consumption [5], profound penetrability [6], and other commendable characteristics [7,8,9]. In practical applications, the meticulous regulation of terahertz wave parameters stands as an imperative pursuit. Among these parameters, the amplitude modulation of terahertz waves utilizing surface plasmon resonance (SPR) metamaterial absorbers constitutes an inherently captivating research realm [10,11,12]. In various fields such as biochemical identification, environmental toxin detection, and safety assessment, the resonant frequencies of most biological molecules fall within the THz range, making THz absorbers more sensitive. They can provide detailed information about the structure and dynamic behavior of biological molecules, such as proteins, DNA, and cell membranes. In contrast, the frequency range of visible-light absorbers and infrared absorbers cannot achieve the sensitivity of the THz range. Moreover, THz absorbers operate at lower frequencies, which can prevent background and substrate absorption [13]. Furthermore, THz waves exhibit strong penetration capabilities, allowing them to penetrate many non-metallic and non-aqueous biological samples, such as cells and tissues. This enables non-destructive biological detection in the THz range, without the need for special sample treatment or the addition of labeling substances, avoiding detrimental effects on biological samples. Visible-light absorbers and infrared absorbers, due to their shorter wavelengths, have poorer penetration capabilities and typically require sample processing or the use of microscopy equipment for detection. Additionally, THz radiation has no significant damaging effects on biological systems, as it has low radiation energy and high biological safety. In contrast, visible-light absorbers and infrared absorbers may cause thermal damage or chemical reactions in biological systems under high-energy radiation. In summary, THz absorbers have a higher sensitivity, a wider range of applications, and greater potential compared to visible-light and infrared absorbers [14,15,16].

However, conventional configurations of metamaterial absorbers are beset by inherent constraints. Once established, adaptability becomes a challenge. For example, some notable references on the subject include metal absorbers [17,18], anisotropic plasmonic metasurfaces [19], and lithography-free metasurface absorbers [20]. Fixed absorptivity and resonance frequencies, accompanied by polarization dependencies and susceptibility to incident angles, collectively confine their pragmatic versatility. Hence, there is a compelling quest for a metamaterial absorber design capable of realizing tunable absorption performance, polarization independence, resistance to variations in incident angles, and exceptional sensing capabilities. In the pursuit of these goals, researchers have harnessed an array of diverse metamaterials, encompassing noble metals [21], graphene [22], vanadium dioxide [23], and Dirac semimetals [24]. Notably, graphene has risen to prominence as a standout candidate, owing to its unparalleled electrical conductivity and carrier mobility [25]. This material engenders swift spectral responses spanning the entire terahertz spectrum [26]. Employing geometric patterning, it incites robust localized surface plasmon resonance (LSPR) [27], enabling the achievement of multiple super-absorption bands and heightened absorption rates. Further enhancing its potential, the dynamic manipulation of graphene’s Fermi energy level through applied voltage facilitates real-time adjustments to the device’s absorption frequency and absorbance characteristics [28,29]. This distinctive capability empowers graphene-based SPR metamaterial absorbers to achieve impeccable absorption at chosen resonant frequencies. This confluence of exceptional attributes positions graphene-based SPR metamaterial absorbers as highly promising contenders for catalyzing pioneering advancements in the manipulation and absorption of terahertz waves.

As for the field of narrowband graphene-based SPR metamaterial absorbers, numerous designs have been reported. For instance, in 2019, Yan et al., explored the impact of Fermi level variation on resonance frequency in a tunable single-mode absorber, achieving an absorption rate of up to 99.99% at a Fermi level of 0 eV [30]. In 2022, Zhu et al., devised an absorber exhibiting both single-band and dual-band absorption characteristics: a 99.30% absorption rate was achieved at 16.0 THz; a 94.56% dual-band absorption rate was observed at 11.4 THz; and a 99.11% absorption rate occurred at 26.2 THz [31]. In 2023, Lai et al., reported a tripe-band SPR metamaterial absorber based on open-ended prohibited sign type mono-layer graphene, achieving three absorption peaks at 4.04 THz, 6.76 THz, and 9.40 THz [32]. Nonetheless, the majority of research has primarily concentrated on one to three absorption peaks in graphene SPR metamaterial absorbers. There remains a scarcity of research reports concerning four or more absorption bands.

In this study, a novel THz graphene-based SPR metamaterial absorber is introduced. Numerical simulations reveal the emergence of penta-band absorption peaks under TE polarization at the frequencies *f*_I_ = 1.99 THz (95.82%), *f*_Ⅱ_ = 6.00 THz (98.47%), *f*_Ⅲ_ = 7.37 THz (98.72%), *f*_IV_ = 8.47 THz (99.87%), and *f*_V_ = 9.38 THz (97.20%). This absorption closely aligns with that observed under TM polarization. Subsequently, a detailed analysis of the absorptivity mechanism is carried out, employing transmission line theory and the equivalent impedance matching theory, supplemented by the visualization of surface electric field distribution diagrams. Moreover, the dynamic tunability of the absorber is showcased by manipulating the Fermi level and relaxation time of the graphene metasurface. Additionally, the influence of geometric parameters within the graphene pattern on absorptivity is studied. The findings underscore the robustness of the graphene’s geometry, particularly concerning its capacity to sustain resonance frequencies. Furthermore, the absorber’s absorptivity is evaluated across a range of incident angles. The results suggest that the device effectively sustains significant absorption efficiency for both TE polarization in the range of 0° to 50° and TM polarization spanning from 0° to 40°. Finally, the absorber’s potential for refractive index sensing within environmental contexts is demonstrated. The results suggest that the maximum sensitivity and FOM are *S*_V_ = 1.75 THz/RIU and FOM_V_ = 12.774 RIU^−1^.

## 2. Structure and Design

**Figure 1:**
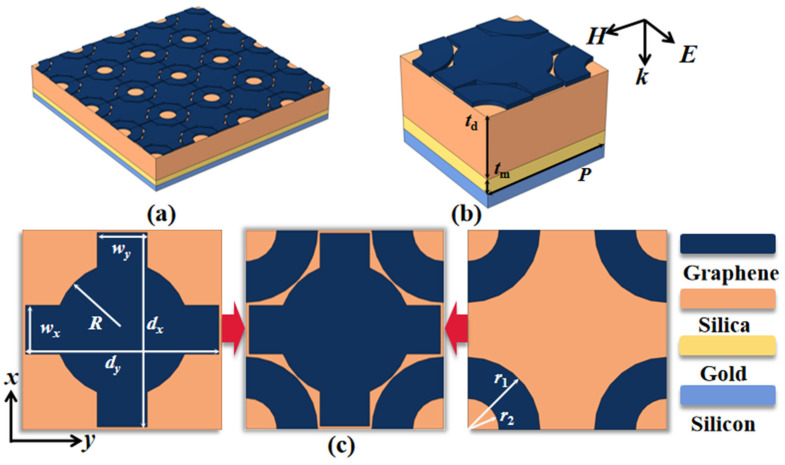
(**a**) A comprehensive view of the device; (**b**) a 3D representation of the unit structure; (**c**) a top-down perspective of the graphene unit pattern, comprising a Celtic cross and a ring divided into four sections.

The specific structure of the device is depicted in Figure 1, showcasing the detailed geometric parameters of our design. All parameters are outlined in Table 1. In this study, the graphene pattern comprises a Celtic cross and a ring divided into four sections. Gold (Au) serves as a reflector to impede the transmission of terahertz waves. Consequently, the thickness *t*_m_ = 1 μm is significantly greater than the skin depth. The device is deposited onto a silicon (Si) substrate.

When an electromagnetic wave impacts the device, the graphene surface becomes excited, giving rise to the generation of surface plasmon waves (SPWs). If the frequency, wave vector, and propagation direction of the electromagnetic wave align with those of the SPW, the energy carried by photons is transferred to the free electrons of the SPW [33]. This phenomenon leads to surface plasmon resonance (SPR) [34]. Consequently, the device effectively captures the energy of the electromagnetic wave. The absorption characteristics of the device are characterized in the following manner [35]:

(1) A=1−R−T

(2) $$R={\left|S_{11}\right|}^{2}$$

(3) $$T={\left|S_{21}\right|}^{2}$$

Here, *R* denotes reflectance, and *T* signifies transmittance. These quantities can also be represented using the *S* parameters, specifically the reflectance rate *S*_11_ and the transmittance rate *S*_21_. In order to achieve *T* = |*S*_21_|^2^ = 0, the thickness of the Au layer must surpass its skin depth to inhibit the transmission of incident light. The skin depth of Au is defined as the distance at which the amplitude of the electromagnetic wave has attenuated to 1/*e* (approximately 37%) of its value at the surface [36]:

(4) $$e^{-\alpha \delta }=1/e\Rightarrow \delta =\frac{1}{\alpha }=\sqrt{\frac{2}{\omega \mu \sigma }}=\frac{1}{\sqrt{\pi f\mu \sigma }}$$

In this study, for gold (Au), the values used are *μ* = *μ*_0_ = 4*π* × 10^−7^ N/A^2^ and *σ* = 4.56 × 10^7^ S/m. Consequently, the maximum skin depth within the frequency range of *f* (1~10 THz) is calculated to be 7.4531 × 10^−2^ μm, which is much smaller than *t*_m_ (1 μm).

In this context, transmission line theory and equivalent impedance matching theory are employed to analyze the objective of achieving *R* = |*S*_11_|^2^ = 0. According to the equivalent impedance matching theory, the equivalent impedance of the device is described as follows [37]:

(5) $$Z_{\mathrm{eff}}=\sqrt{\frac{{\left(1+S_{11}\right)}^{2}-S_{21}^{2}}{{\left(1-S_{11}\right)}^{2}-S_{21}^{2}}}=\frac{1+S_{11}}{1-S_{11}}(S_{21}=0)$$

Therefore, perfect absorption is achieved when *S*_11_ = 0, *Z*_eff_ = 1, that is, when Re(*Z*_eff_) = 1 and Im(*Z*_eff_) = 0. In fact, *S*_11_ and *R* can be expressed according to Equation (5) as follows:

(6) $$S_{11}=\frac{Z_{\mathrm{eff}}-1}{Z_{\mathrm{eff}}+1}=\frac{Z_{\mathrm{eff}}\cdot Z_{0}-Z_{0}}{Z_{\mathrm{eff}}\cdot Z_{0}+Z_{0}}=\frac{Z_{\mathrm{in}}-Z_{0}}{Z_{\mathrm{in}}+Z_{0}}$$

(7) $$R={\left(\frac{Z_{\mathrm{in}}-Z_{0}}{Z_{\mathrm{in}}+Z_{0}}\right)}^{2}$$

Here, *Z*_in_ = *Z*_eff_·*Z*_0_ signifies the input impedance of the graphene layer, where *Z*_0_ represents the intrinsic impedance of free space. When *Z*_in_ equals *Z*_0_, indicating that the input impedance of the absorber matches the intrinsic impedance of free space, perfect absorption can be attained [38]. Equation (7) can be elucidated utilizing transmission line theory.

**Figure 2:**
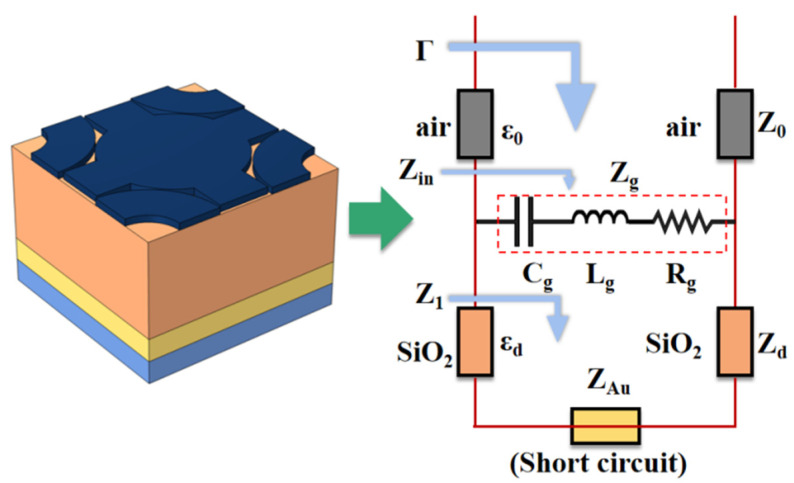
The equivalent circuit model of the absorber.

An equivalent circuit model can be established for the device [39], as shown in Figure 2. The absorber is equivalent to an RLC series circuit. Since the gold layer only functions to reflect the electromagnetic wave during absorption, it can be treated as a short circuit [40]. The impedance of the silicon dioxide layer and its input impedance are expressed as follows [41]:

(8) $$\left\{ \begin{array}{l} Z_{1}=jZ_{d}\tan(k_{d}t_{d})\\ Z_{d}=\sqrt{\mu_{0}/\varepsilon_{0}\varepsilon_{d}}\\ k_{d}=2\pi f\sqrt{\varepsilon_{0}\varepsilon_{d}\mu_{0}} \end{array}\right.$$

The impedance of the graphene layer is given by

(9) $$Z_{g}=R_{g}+jX_{g}=R_{g}+j(2\pi fL_{g}-\frac{1}{2\pi fC_{g}})$$

When SPR occurs at a certain incident electromagnetic wave frequency, the entire circuit is in a resonant state, exhibiting resistive behavior. Therefore,

(10) $$X_{g}=0\Rightarrow Z_{g}=R_{g}$$

At this point, the input impedance of it is as follows:

(11) $$Z_{\mathrm{in}}=\frac{Z_{1}\cdot Z_{g}}{Z_{1}+Z_{g}}$$

The input impedance of the circuit is

(12) $$\left\{ \begin{array}{l} \Gamma =\frac{Z_{\mathrm{in}}-Z_{0}}{Z_{\mathrm{in}}+Z_{0}}=S_{11}\\ Z_{0}=\sqrt{\frac{\mu_{0}}{\varepsilon_{0}}}(Z_{0}=120\pi \approx 377\Omega ) \end{array}\right.$$

Hence, *A* = 1 − *R* can be described as follows: only when *Z*_in_ = *Z*_0_, *A* reaches its maximum value.

(13) $$A=1-R=1-{\left|S_{11}\right|}^{2}=1-\Gamma^{2}=1-{\left(\frac{Z_{\mathrm{in}}-Z_{0}}{Z_{\mathrm{in}}+Z_{0}}\right)}^{2}=\frac{4}{\frac{Z_{\mathrm{in}}}{Z_{0}}+\frac{Z_{0}}{Z_{\mathrm{in}}}+2}$$

In conclusion, achieving perfect absorption through the alignment of the absorber’s input impedance with the intrinsic impedance of free space necessitates a meticulous choice of several key factors. These factors include the incident frequency (*f*), the dielectric layer’s material (*ε*_d_) and thickness (*t*_d_), as well as the impedance of graphene (*Z*_g_). In terms of the dielectric layer, silica was selected due to its exceptional hardness and resistance to abrasion, which render it particularly well suited to the demanding operational conditions. The determination of the appropriate thickness for this layer will be explored in the subsequent section. The impedance of graphene is intricately tied to its relative permittivity, which is expressed as follows [42]:

(14) $$\varepsilon_{g}=1+j\frac{\sigma_{g}(\omega )}{\varepsilon_{0}\omega t_{g}}$$

where *t*_g_ represents the thickness of the graphene layer. In this study, a value of *t*_g_ = 1 nm was selected for ease of calculation purposes.

The total surface conductivity of graphene is defined as follows [43]:

(15) $$\sigma_{g}(\omega )=\sigma_{\mathrm{inter}}(\omega )+\sigma_{\mathrm{intra}}(\omega )$$

where *σ*_inter_(*ω*) denotes the interband conductivity of graphene, and *σ*_intra_(*ω*) represents the intraband conductivity. These can be expressed using the classical Kubo formula [44]:

(16) $$\sigma_{\mathrm{inter}}(\omega )=\frac{ie^{2}}{4\pi \hbar^{2}}\ln\left[\frac{2\left|E_{F}\right|-\hbar (\omega +i\tau^{-1})}{2\left|E_{F}\right|+\hbar (\omega +i\tau^{-1})}\right]$$

(17) $$\sigma_{\mathrm{intra}}(\omega )=\frac{ie^{2}k_{B}T}{\pi \hbar^{2}(\omega +i\tau^{-1})}\left\{\frac{E_{F}}{k_{B}T}+2\ln\left[\exp(-\frac{E_{F}}{k_{B}T})+1\right]\right\}$$

where

*e* represents the elementary charge,

ℏ is the reduced Planck constant,

*E_F_* signifies the Fermi energy level of graphene,

*ω* stands for the angular frequency of the incident electromagnetic wave,

*τ* is the relaxation time of graphene,

*k*_B_ denotes the Boltzmann constant,

*T* represents the ambient temperature in Kelvin.

According to the Pauli exclusion principle, within the terahertz range, where *E_F_* is much greater than ℏω, graphene’s surface conductivity is primarily determined by intraband effects. As a result, the contribution of the interband conductivity *σ*_inter_(*ω*) can be disregarded, leading to the simplification of *σ*_g_(*ω*) using the Drude model [45] as follows:

(18) $$\sigma_{g}(\omega )=\sigma_{\mathrm{intra}}(\omega )=\frac{ie^{2}\left|E_{F}\right|}{\pi \hbar^{2}(\omega +i\tau^{-1})}$$

**Figure 3:**
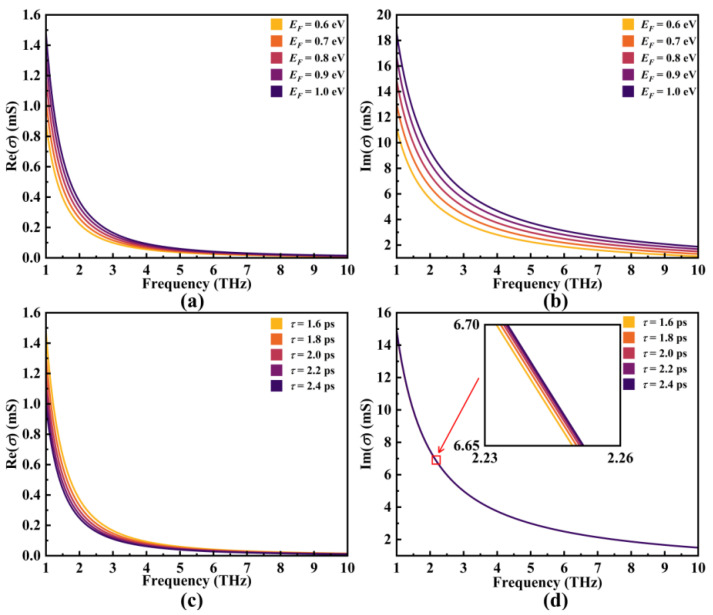
(**a**,**b**) The correlation between Re(*σ*) and Im(*σ*) with respect to the Fermi level (*E_F_*) of graphene when *τ* = 2.0 ps; (**c**,**d**) the connection between Re(*σ*) and Im(*σ*) in relation to the relaxation time (*τ*) when *E_F_* = 0.8 eV.

Based on the discussions presented above, it is evident that *σ*_g_(*ω*) can be modulated by manipulating the frequency of incident light (*ω* or *f* = 2*πω*), the Fermi level (*E_F_*), and the relaxation time (*τ*) of graphene. These dynamics are visually depicted in Figure 3, showcasing how graphene’s surface conductivity changes with respect to the incident frequency and *E_F_* at *τ* = 2.0 ps, as well as concerning the incident frequency and *τ* at *E_F_* = 0.8 eV. This provides a theoretical foundation for subsequent simulation results.

## 3. Simulation Process

This section will explain the building of the proposed simulation model of a graphene terahertz absorber in COMSOL Multiphysics 5.6 software.

Following the construction of the model using the parameters listed in Table 1, an analyte, such as air, was added above the model. The thickness of the analyte is recommended to be greater than 30 μm. Next, the corresponding material parameters for each geometric part were added. The silicon substrate was not created during the simulation.

In the x–y plane, periodic boundary conditions were implemented, while perfect match layers were established in the z-direction to absorb the excess scattered waves. The transition boundary condition was applied to the graphene layer. The incident electromagnetic wave frequency was set between 1 and 10 THz. The excitation port was set above the analyte to simulate the incident electromagnetic wave, and the port was set below the gold layer to extract the S parameters. The “Type of port” was selected as “Periodic”.

For meshing, the sequence type was configured as “User-controlled mesh”. A new “Free triangular” mesh was created for the graphene layer, with the specified “Size” parameters. The “Maximum element size” was set to 0.2 μm, and the “Minimum element size” was set to 0.1 μm. The remaining meshing settings were left as default.

## 4. Simulation Results and Discussion

**Figure 4:**
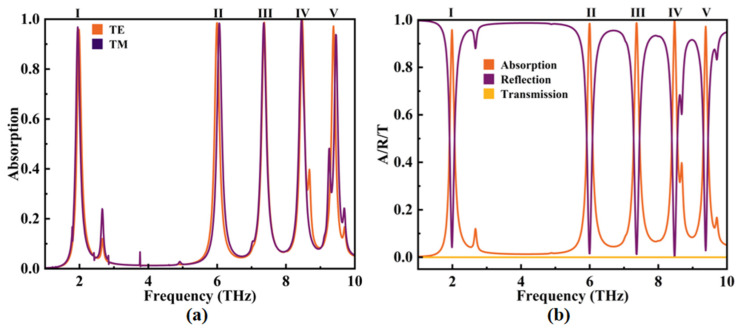
(**a**) The absorption rate diagrams of the device under TE polarization and TM polarization. (**b**) The A/R/T graph of the absorber under TE polarization.

Distinct tests were conducted to evaluate the absorption performance of the absorber under both TE and TM polarizations, as depicted in Figure 4a. Five absorption peaks, designated as Modes Ⅰ–V, are clearly discernible. Notably, apart from Mode V wherein a slight variance in absorption rate and resonance frequency is observed, the other modes remain essentially unaffected. This underscores the polarization independence of the absorber’s absorption characteristics. Unless otherwise stipulated, all discussions in this study are grounded in TE polarization.

**Figure 5:**
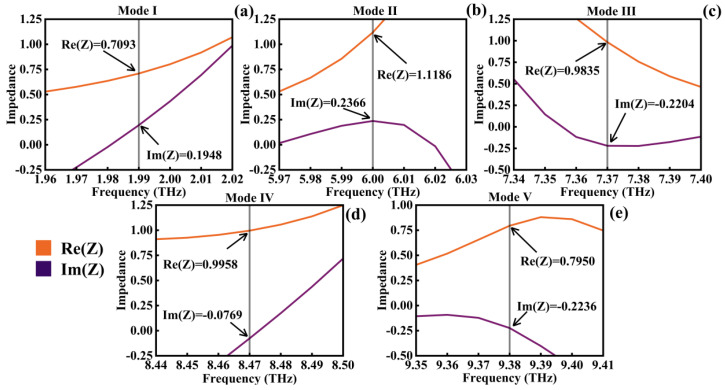
Equivalent impedance matching diagrams for Modes I−V (**a**−**e**).

The corresponding equivalent impedance matching diagrams for each mode were mapped as shown in Figure 5. It can be observed that, at these resonance frequencies, Re(*Z*) is close to 1 and Im(*Z*) is close to 0, especially for Mode IV (Figure 5d) with an absorption rate of 99.87%, Re(*Z*) = 0.9958, and Im(*Z*) = −0.0769, indicating a remarkably close approximation to perfect absorption. These experimental results align very closely with the theoretical derivations discussed earlier.

**Figure 6:**
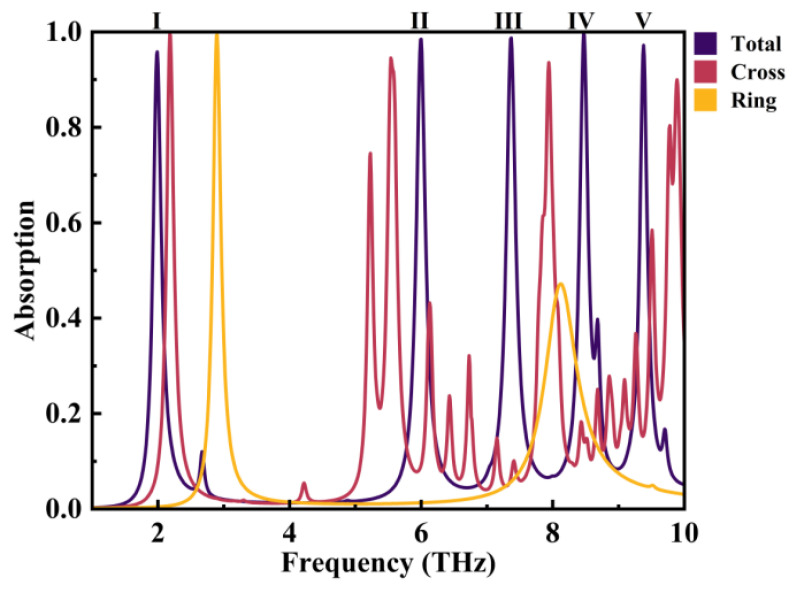
The absorption rate diagram of the device includes the overall absorption rate diagram of the entire geometric structure, along with absorption rate diagrams for two subpatterns.

As illustrated in Figure 6, the total absorption rate diagram of the device arises from the coupling of two subpatterns. It is evident that significant SPR occurs at five different resonant frequencies: *f*_I_ = 1.99 THz, *f*_Ⅱ_ = 6.00 THz, *f*_Ⅲ_ = 7.37 THz, *f*_IV_ = 8.47 THz, and *f*_V_ = 9.38 THz. The corresponding absorption rates for these modes were 95.82%, 98.47%, 98.72%, 99.87%, and 97.20%, respectively.

**Figure 7:**
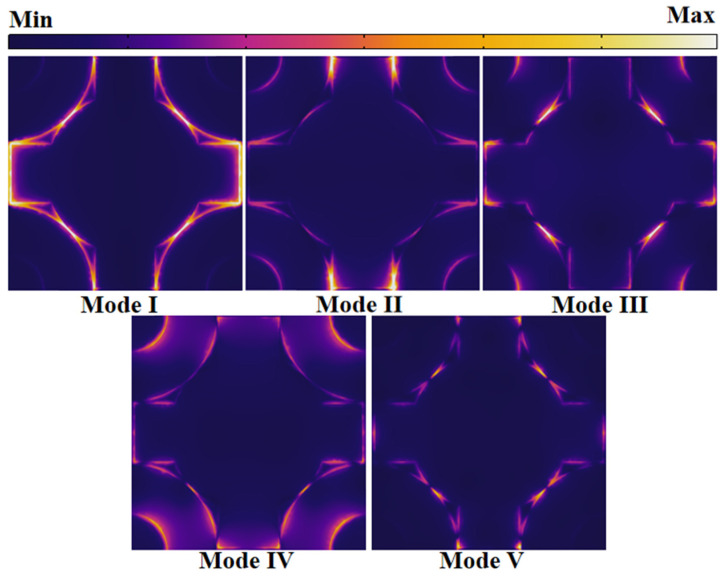
Surface electric field distribution maps of the five modes in the x–y plane.

To ascertain the precise positions at which LSPR occurs on the graphene patterns for each mode, electric field monitors were strategically positioned on the x–y plane. The resultant findings are visually depicted in Figure 7.

In Mode Ⅰ, localized surface plasmon resonance (LSPR) was predominantly concentrated in the proximity of the ring and the Celtic cross, extending along the cross’s edges in the y-direction. This behavior can be attributed to the coupling with neighboring periodic graphene patterns. Furthermore, regions along the ring’s edges exhibited a relatively weaker LSPR intensity. In Mode II, robust resonance was localized near the Celtic cross’s edges in the x-direction and the adjacent region of the ring. In contrast, other positions displayed subdued responses. Moreover, a relatively weaker surface plasmon resonance was discernible along the inner edge of the ring.

In Mode III, significant LSPR contributions were evident near the circular segment of the Celtic cross, particularly in proximity to the ring. Additionally, pronounced LSPR effects were observed along the Celtic cross’s edges in the y-direction. For Mode IV, LSPR was conspicuous across several positions of the pattern, primarily along both sides of the ring’s edges and the edges of the Celtic cross, coupled with adjacent periodic patterns.

In Mode V, LSPR predominantly emerged within the gaps between the Celtic cross and the ring. These gaps encompass the spaces between the circle and the ring, between the Celtic cross and the ring, and between the Celtic cross and the adjacent periodic cross.

Owing to the inherent symmetry of the graphene pattern, the regions in which LSPR is excited maintain symmetry as well [46]. This characteristic enhances the absorber’s capability to sustain absorption performance across a broader range of incident angles. Throughout this paper, unless explicitly stated, all discussions are predicated on the scenario of normal electromagnetic wave incidence, where the incident angle (*θ*) is set at 0 degrees. Notably, in this study, it became apparent that the gaps between the subpatterns and their edges played a pivotal role in the occurrence of surface plasmon resonance [47,48]. Therefore, it is imperative to manipulate the geometric parameters of these regions to explore how variations impact the absorber’s absorption performance.

**Figure 8:**
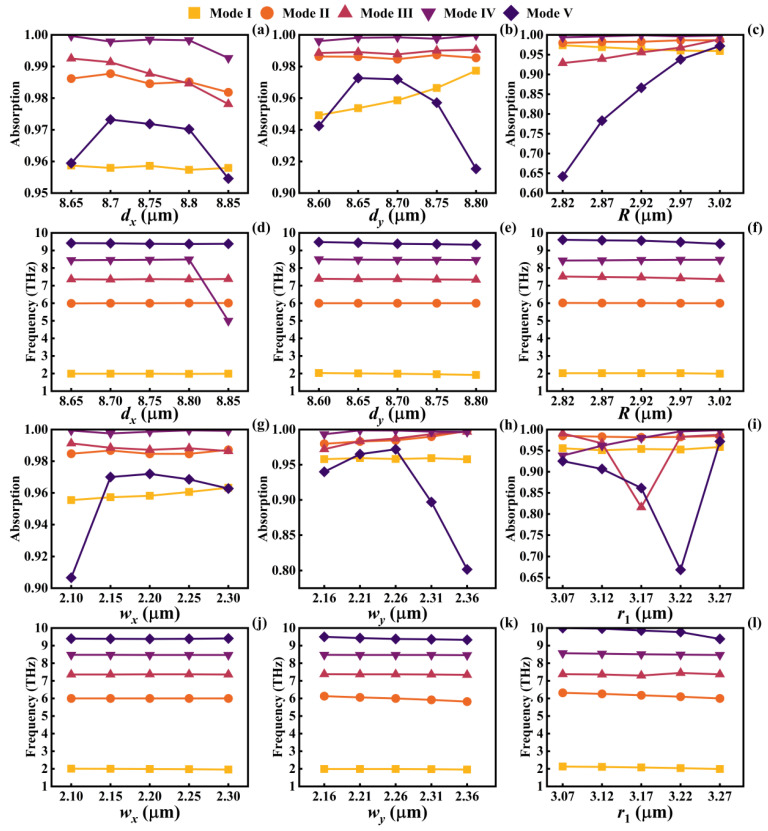
The impact of variations in geometric parameters on the absorber’s absorption rates (**a**−**c**,**g**−**i**) and resonance frequencies (**d**−**f**,**j**−**l**).

Numerical analyses were performed on the geometric parameters of the graphene pattern (excluding *r*_2_, as it scales with *r*_1_). Hence, this study focused solely on variations in the overall size of the ring. And the results are depicted in Figure 8. Overall, the alteration of various geometric parameters did not significantly shift the resonant frequencies of the five modes. This indicates that the geometric parameters of the graphene pattern exhibit robustness in affecting resonance frequencies. However, changing the geometric parameters can lead to relatively significant variations in the absorption rate. In summary, in this study, the gaps between subpatterns emerged as crucial in generating SPR and achieving high absorption rates. One of the techniques for designing multiband narrowband metamaterial absorbers involves the thoughtful design of geometric patterns on the metasurface, creating multiple coupling regions. If the frequencies at which SPR occurs are continuous, maintaining elevated absorption rates across a range of frequencies, it becomes possible to devise a wideband SPR metamaterial absorber [49,50]. The geometric parameters of a graphene metasurface play a significant role in influencing the absorption of light based on the incident angle or polarization state. For instance, a graphene surface with high symmetry can maintain a consistently high absorption rate across a wide range of incident angles. Adjusting the geometric parameters allows for narrowing the absorption angle range or shifting the resonance frequency. Additionally, a graphene metasurface with a chiral pattern can selectively absorb light waves of a specific polarization state, and modifying the geometric parameters can alter its polarization selectivity. Modifying the geometric parameters directly influences the surface conductivity of graphene, consequently altering the impedance of the absorber. Inadequate geometric design can lead to suboptimal impedance matching, resulting in unsatisfactory absorption performance. On the other hand, well-designed geometric parameters not only enhance the robustness of the graphene pattern but also enable effective impedance matching within a specific resonance frequency range. Additionally, they ensure broad-angle absorption capabilities.

**Figure 9:**
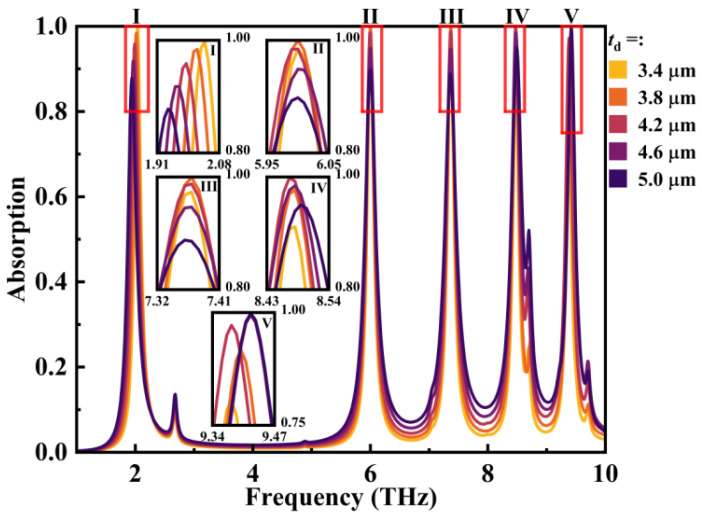
The impact of the thickness of the SiO_2_ layer on absorptivity of the device.

Furthermore, the impact of the thickness of the silica layer on absorption performances was tested, as depicted in Figure 9. At lower frequencies, reducing the thickness of the silica layer enhanced the absorption rate. Conversely, at higher frequencies, it is advisable to avoid an excessively thick silicon dioxide layer. This rule can be found in reference [51]. The graphene layer and the gold layer form an asymmetric Fabry–Perot cavity. Quoting the formula, the total reflection coefficient of the device can be expressed as follows:

(19) $$\begin{array}{l} r=r_{12}+r_{m}=r_{12}+\frac{t_{12}t_{21}r_{23}e^{\left(-2i\phi \right)}}{1-r_{21}r_{23}e^{\left(-2i\phi \right)}}\\ \phi =k_{0}nd\cos\theta ’ \end{array}$$

where *d* presents the thickness of the dielectric layer (*t*_d_ in this paper). When light is incident on the absorber, the graphene surface generates LSPR, and the light waves undergo multiple reflections within the Fabry–Perot cavity. By carefully selecting the appropriate material and thickness for the dielectric layer, the interference of the reflected light from different orders can be effectively suppressed. This leads to the occurrence of Fabry–Perot resonance, where the Fabry–Perot resonance and the LSPR on the graphene surface are strongly coupled. As a result, the electromagnetic waves are efficiently absorbed. After multiple experiments, it was determined that the average highest absorption rate for the five modes was achieved when *t*_d_ = 4.2 μm.

**Figure 10:**
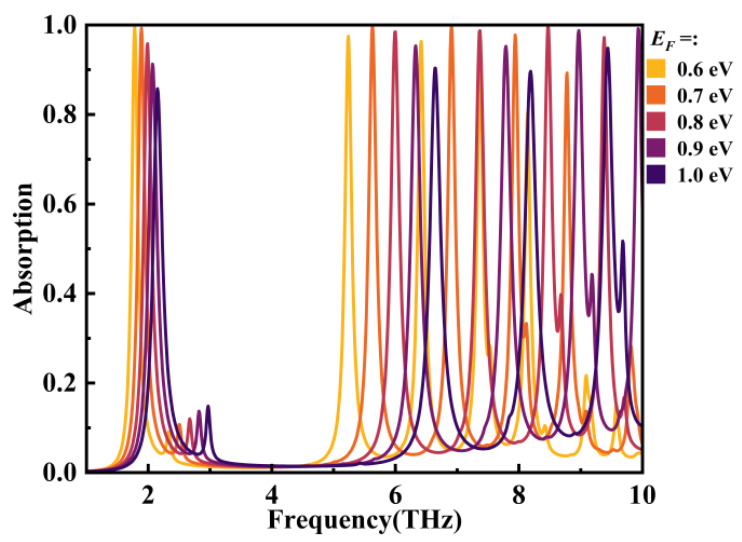
The impact of variations in *E_F_* under *τ* = 2.0 ps on the absorber’s absorption performance.

Next, the dynamic tunability of the device was demonstrated by varying the Fermi level (*E_F_*) and relaxation time (*τ*) of graphene. The effect of varying the Fermi level (*E_F_*) under a relaxation time (*τ*) of 2.0 ps on the absorption performance of the absorber was initially examined. As shown in Figure 10, it is evident that with increasing *E_F_*, the resonance frequencies of the five modes all experienced a blue shift. This shift occurs because the wavevector of surface plasmon polaritons (SPPs) satisfies the following relationship [52]:

(20) $$k_{\mathrm{spp}}\propto \hbar f_{r}^{2}/\left(2\alpha_{0}E_{F}c\right)$$

where *α*_0_ is fine structure constant and *f_r_* is resonance frequency. Hence, there exists a relationship between the Fermi level and resonance frequency:

(21) $$f_{r}\propto E_{F}^{1/2}$$

Therefore, the resonance frequency will increase with an increase in *E_F_*, and the modulation bandwidth at higher frequencies will also be larger. There is a relationship between the Fermi level and the applied voltage [53],

(22) $$E_{F}=V_{F}\sqrt{\frac{\pi \varepsilon_{0}\varepsilon_{r}V_{g}}{e_{0}t_{d}}}$$

where

*V_F_* is the Fermi velocity, with a typical value of approximately 10^6^ m/s,

*ε*_0_ and *ε_r_* are the vacuum permittivity and relative permittivity, respectively,

*V*_g_ is the applied external voltage,

*e*_0_ is the elementary charge,

*t*_d_ is the thickness of the dielectric (SiO_2_) layer.

It is apparent that, by maintaining a constant thickness of the dielectric layer, the magnitude of *E_F_* can be controlled through the manipulation of the applied external voltage (*V*_g_). In practical applications, once the device is fabricated, its absorption performance remains fixed. By applying a gate voltage, the capacity to dynamically adjust the Fermi level of graphene is gained, thereby influencing its conductivity. This dynamic capability allows the device to dynamically fine-tune both the absorption rate and resonance frequency, thereby ensuring optimal performance.

**Figure 11:**
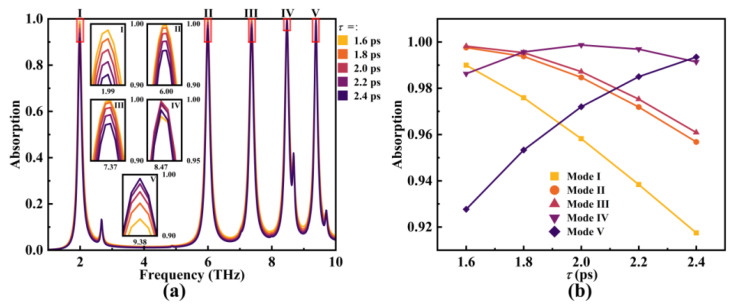
The influence of *τ* variation on the resonant frequency (**a**) and absorption performance (**b**) of the absorber under the condition of *E_F_* = 0.8 eV.

Subsequently, the changes in absorber performance were examined in relation to the relaxation time (*τ*) at a fixed Fermi level (*E_F_*) of 0.8 eV. The outcomes of this investigation are depicted in Figure 11. As *τ* increased from 1.6 ps to 2.4 ps, the resonance frequencies of each mode remained constant, with only the absorption rate being modulated. This observation is consistent with the connection between the relaxation time of graphene and the Fermi level, as illustrated by the following relationship [54]:

(23) $$\tau =E_{F}v/(eV_{F}^{2})$$

where *v* represents the carrier mobility of graphene. With *τ* increasing from 1.6 ps to 2.4 ps, the resonant frequencies of each mode remained unaffected, while only the absorption rate underwent modulation. Since *E_F_* remains constant, alterations in *τ* induce variations in the concentration of graphene carriers, subsequently influencing the intensity of plasmonic oscillations [55,56]. This cascade effect ultimately leads to changes in the absorption rate. In conclusion, the manipulation of graphene’s Fermi level and relaxation time allows for effective control over the absorption rate and resonance frequency of graphene-based SPR metamaterial absorbers. In comparison to noble metal SPR metamaterial absorbers, our device exhibits the potential for broader practical applications.

According to the Fresnel equations, the transverse electric and transverse magnetic reflectances of the metamaterial absorber are, respectively, expressed as follows [57,58]:

(24) $$\left\{ \begin{array}{l} R_{\mathrm{TE}}={\left|\frac{\cos\theta -\mu_{r}^{-1}\sqrt{n^{2}-\sin^{2}\theta }}{\cos\theta +\mu_{r}^{-1}\sqrt{n^{2}-\sin^{2}\theta }}\right|}^{2}\\ R_{\mathrm{TM}}={\left|\frac{\varepsilon_{r}\cos\theta -\sqrt{n^{2}-\sin^{2}\theta }}{\varepsilon_{r}\cos\theta +\sqrt{n^{2}-\sin^{2}\theta }}\right|}^{2} \end{array}\right.$$

(25) $$n=\sqrt{\varepsilon_{r}\mu_{r}}$$

where *θ* represents the incident angle of the electromagnetic wave, and *n* denotes the effective refractive index of the metamaterial absorber. Taking TE polarization as an example, when the electromagnetic wave is normally incident, that is, *θ* = 0°, the expression for *R*_TE_ in Equation (23) can be written as follows:

(26) $$R_{\mathrm{TE}}={\left|\frac{\sqrt{\mu_{r}}-\sqrt{\varepsilon_{r}}}{\sqrt{\mu_{r}}+\sqrt{\varepsilon_{r}}}\right|}^{2}={\left|\frac{Z-Z_{0}}{Z+Z_{0}}\right|}^{2}$$

**Figure 12:**
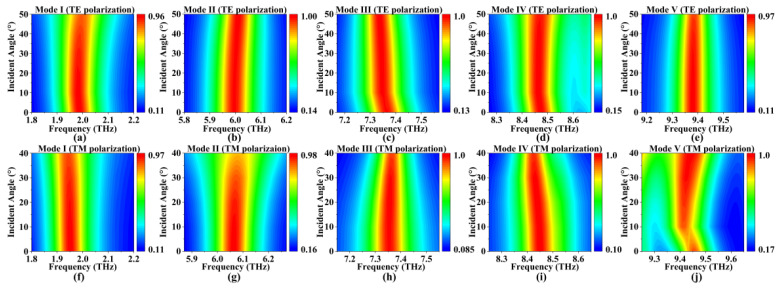
(**a**–**e**) The variation in the absorption performance with incident angle under TE polarization; (**f**–**j**) the changes in absorptivity with incident angle under TM polarization.

This finding correlates with Equation (7). Moreover, Equation (23) emphasizes that the effect of graphene’s impedance is not solely limited to polarization modes; the incident angle of the electromagnetic wave also holds significant influence. Consequently, it is essential to conduct a thorough investigation into how the incident angle affects the absorption performance of the absorber. To address this, an incident angle ranging from 0° to 50° for TE polarization and from 0° to 40° for TM polarization was set up. The simulation outcomes are graphically presented in Figure 12.

Under TE polarization, as the incident angle varied from 0 to 50 degrees, slight shifts in resonant frequencies were observed for each mode, while the absorption rates consistently remained above 85%. Similarly, in the case of TM polarization, in which the incident angle spanned from 0 to 40 degrees, each mode displayed minor resonant frequency shifts, while the absorption rates consistently stayed above 82%. This observation underscores the device’s capability to maintain substantial absorption rates even at larger incident angles. This phenomenon can be attributed to two key factors. Firstly, the device’s dimensions are smaller than the vacuum wavelength of the incident light, allowing it to effectively interact with a wide range of angles [59,60]. Additionally, the inherent symmetry of the graphene patterns plays a crucial role. Within a specific range of incident angles, the symmetry of the graphene patterns remains intact, ensuring robust SPR at the corresponding positions as illustrated in Figure 7.

In summation, our design exhibited remarkable insensitivity to incident angles, encompassing a range from 0 to 50 degrees in the TE mode and 0 to 40 degrees in the TM mode. For practical applications, the incorporation of concave structures positioned above the absorber’s surface could be explored [61,62]. This strategic approach could facilitate the coupling of incident light with the graphene metasurface at specific angles, thus ensuring a consistently high absorption rate.

**Figure 13:**
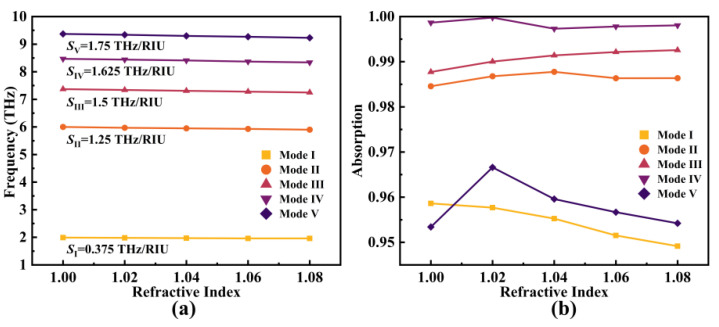
The variation in resonant frequencies (**a**) and absorption rates (**b**) of each mode as the ambient refractive index changes from 1.00 to 1.08.

A pivotal performance aspect of the metamaterial SPR absorber is its capability for refractive index (RI) sensing. An analyte refractive index (*n*) range of 1.00 to 1.08 was selected, and the resulting shifts in the resonant frequencies and absorption rates were explored. The outcomes are visualized in Figure 13. Notably, as the refractive index increased, all five modes exhibited a pronounced red-shift in their resonant frequencies, with their absorption rates consistently maintained above 94.9%.

The ranges of variation for these shifts were Δ*f*_I_: 1.96~1.99 THz, Δ*f*_Ⅱ_: 5.9~6 THz, Δ*f*_Ⅲ_: 7.25~7.37 THz, Δ*f*_IV_: 8.34~8.47 THz, and Δ*f*_V_: 9.23~9.37 THz, respectively. The sensitivity *S* is defined as follows [63]:

(27) $$S=\frac{\Delta f}{\Delta n}$$

where Δ*n* was 0.08 in this research. Hence, the corresponding *S* for each mode were *S*_I_ = 0.375 THz/RIU, *S*_II_ = 1.25 THz/RIU, *S*_III_ = 1.5 THz/RIU, *S*_IV_ = 1.625 THz/RIU, and *S*_V_ = 1.75 THz/RIU, respectively. The average sensitivity was *S*_avg_ = 1.3 THz/RIU, where RIU refers to the refractive index unit. According to the obtained results, the device was highly responsive to changes in the refractive index of the analyte, making it suitable for deployment as a refractive index sensor (RI sensor).

**Figure 14:**
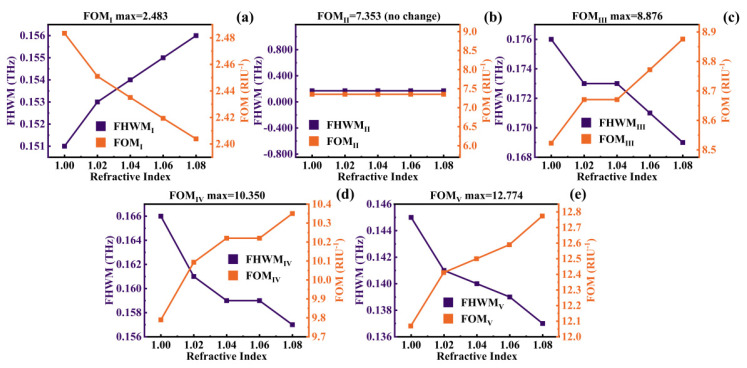
The variation in the FWHM and FOM of Mode I–V (**a**–**e**) with *n* ranges from 1.00 to 1.08.

The figure of merit (FOM) is another crucial factor for assessing the sensing performance of the absorber. It is defined as follows [64,65]:

(28) $$\mathrm{FOM}=\frac{S}{\mathrm{FHWM}}$$

where FHWM represents the full width at half maximum of the absorption peak. As indicated by Equation (27), Table 2 presents the average FHWM and FOM values for the device. Meanwhile, Table 3 and Figure 14 provides insight into the minimum FHWM for each mode along with its corresponding maximum FOM.

From the results, it can be inferred that the absorber achieved a minimum FHWM of 137 GHz and a maximum FOM of 12.774 RIU^−1^. This underscores the absorber’s strong sensing capabilities and resonance frequency selectivity. Lastly, in order to provide a comprehensive assessment of the absorber’s performance, a comparative analysis between the device and other metamaterial SPR absorbers was conducted. The results are presented in Table 4.

It is clear that our design, when compared with previous studies, exhibits a higher number of absorption peaks and a substantial improvement in refractive index sensing performance (FOM). This strongly indicates the device’s considerable potential for application in fields like biochemistry and sensing.

## 5. Conclusions

In summation, this study presents a new SPR terahertz absorber based on a single-layer, patterned graphene metasurface. Through rigorous theoretical exploration and meticulous numerical simulations, the penta-band absorption, dynamic tunability, geometric robustness, polarization independence, wide-angle absorption, and great sensing capacity of the absorber were elucidated. Compared with other works, the device features a simple structure that not only widens the number of absorption peaks but also ensures a high absorption rate and great refractive index sensing performance. Our work promotes diversity in the design of graphene-based metamaterial absorbers, offering fresh design inspiration. Consequently, we believe this novel SPR metamaterial absorber holds promising potential for applications in active optoelectronic devices, modulators, optical stealth, RI sensors, and other related fields.

## Figures and Tables

**Table 1:** Geometric parameters of the device.

Name	*P*	*t*_d_	*t*_m_	*w_x_*	*w_y_*	*d_x_*	*d_y_*	*R*	*A*	*r*_1_	*r*_2_
Value (μm)	9	4.2	1	2.2	2.26	8.7	8.75	3.02	0.43	3.27	*r*_1_∙*A*

**Table 2:** FHWM_avg_ and FOM_avg_ for each mode as RI varies within the range of 1.00 to 1.08.

Mode	I	II	III	IV	V
FHWM_avg_ (GHz)	153.8	170	172.4	160.4	140.4
FOM_avg_ (RIU^−1^)	2.438	7.353	8.703	10.134	12.469

**Table 3:** FHWM_min_ and FOM_max_ for each mode as RI varies within the range of 1.00 to 1.08.

Mode	Ⅰ	II	III	IV	V
FHWM_min_ (GHz)	151	170	169	157	137
FOM_max_ (RIU^−1^)	2.483	7.353	8.876	10.350	12.774

**Table 4:** Comparison with previous studies.

References	[43]	[66]	[32]	[67]	[68]	This Work
Number of absorption peaks	1	1	3	3	5	5
Tunability	Yes	No	Yes	Yes	Yes	Yes
S_max_ (THz/RIU)	1.57	2.1	2.00	1.867	0.066	1.75
FOM_max_ (RIU^−1^)	24.5	7.03	9.58	2.14	N/A	12.774

## Data Availability

Publicly available datasets were analyzed in this study. This data can be found here: https://www.lumerical.com/ (accessed on 1 January 2020).
